# New drug target in protozoan parasites: the role of thioredoxin reductase

**DOI:** 10.3389/fmicb.2015.00975

**Published:** 2015-09-30

**Authors:** Rosa M. Andrade, Sharon L. Reed

**Affiliations:** ^1^Division of Infectious Diseases, Department of Medicine, University of California San DiegoLa Jolla, CA, USA; ^2^Division of Infectious Diseases, Department of Pathology, School of Medicine, University of California San DiegoLa Jolla, CA, USA

**Keywords:** amebiasis, thioredoxin reductase, auranofin, *Entamoeba histolytica*, protozoan, diarrhea

## Abstract

Amebiasis causes approximately 70,000 deaths annually and is the third cause of death due to parasites worldwide. It is treated primarily with metronidazole, which has adverse side effects, is mutagenic and carcinogenic, and emergence of resistance is an increasing concern. Unfortunately, better therapeutic alternatives are lacking. Re-purposing of older FDA approved drugs is advantageous to drug discovery since safety and pharmacokinetic effects in humans are already known. In high throughput screening studies, we recently demonstrated that auranofin, a gold containing compound originally approved to treat rheumatoid arthritis, has activity against trophozoites of *E. histolytica*, the causative agent of amebiasis. Auranofin's anti-parasitic activity is attributed to its monovalent gold molecule that readily inhibits *E. histolytica* thioredoxin reductase. This anti-oxidant enzyme is the only thiol-dependent flavo-reductase present in *E. histolytica*. Auranofin has also shown promising activity against other protozoans of significant public health importance. Altogether, this evidence suggests that auranofin has the potential to become a broad spectrum alternative therapeutic agent for diseases with a large global burden.

## Introduction

The three major causes of protozoal diarrhea worldwide are *E. histolytica, G. lamblia*, and *Cryptosporidium* sp., which cause significant morbidity and mortality in developing and developed countries (Fletcher et al., 2012). In fact, it is estimated that *E. histolytica* infects approximately 500 million people worldwide, resulting in 50 million cases of invasive disease and about 70,000 deaths annually (Debnath et al., 2012). Meanwhile, *G. lamblia* infection prevalence is estimated at 280 million cases annually^1^ while *Cryptosporidium* sp. accounts for about 20% and up to 9% of diarrheal episodes in children from developing (Simango and Mutikani, 2004; Cama et al., 2008) and developed^2^ countries respectively. Although *Cryptosporidium* sp. infection can be self-limited in immunocompetent people, but it is chronic and debilitating in immunosuppressed and malnourished individuals (Cabada and White, 2010). Because of their link with poverty and association with poor cognitive function in early childhood (Berkman et al., 2002), *Giardia* and *Cryptosporidium* were included in the WHO Neglected Diseases Initiative in 2004 (Savioli et al., 2006). Also, the National Institutes of Health (NIH) has listed *G. lamblia, C. parvum*, and *E. histolytica* as category B priority biodefense pathogens because of their low infectious dose and potential for dissemination through compromised food and water supplies in the United States^3^.

Despite their global burden in public health, there are no vaccines or prophylactic medications to prevent amebiasis, giardiasis or cryptosporidiosis. Furthermore, first-line treatment for invasive amebiasis and giardiasis is metronidazole since 1966 (Powell et al., 1967) and 1963 (Lionetto et al., 1963) respectively. Metronidazole has been shown to be both mutagenic in a microbiological system and carcinogenic to rodents and frequently causes gastrointestinal side effects. *In vitro, E. histolytica* trophozoites can adapt to therapeutically relevant levels of metronidazole (Wassmann et al., 1999). Treatment failures in giardiasis occur in up to 20% of cases (Upcroft and Upcroft, 2001). Clinical resistance of *G. lamblia* to metronidazole has been documented along with cross resistance to the newer drugs, tinidazole and nitazoxanide, making drug resistance an increasing concern (Tejman-Yarden et al., 2013). Nitazoxanide, the only FDA-approved drug for the treatment of cryptosporidiosis, is effective in the treatment of immunocompetent patients but only partially effective for immunosuppressed patients. Therefore, it is critical to develop more effective drugs to treat amebiasis, giardiasis and cryptosporidiosis.

Reprofiling of FDA approved drugs is an advantageous approach to drug discovery since safety and pharmacokinetic effects in humans have already been confirmed clinically. These efforts led to the discovery that auranofin, a gold containing compound that is FDA approved for the treatment of rheumatoid arthritis, has anti-parasitic activity (Angelucci et al., 2009) Its anti-parasitic activity, likely stems from the gold molecule that readily dissociates and inhibits thiol- dependent flavoreductases (Saccoccia et al., 2012) such as thioredoxin-thioredoxin reductase and glutathione-glutathione reductase. These latter enzymes are the two main detoxifying systems that are independent of reactive oxygen species (ROS). These antioxidant systems are present in all protozoan parasites which are constantly exposed to ROS from their own metabolism and those from the host (Hirt et al., 2002).

### Thioredoxin reductase and its diversity among protozoan parasites

Like all eukaryotes, protozoan parasites' defense mechanisms include anti-oxidant systems to handle ROS challenges. Among these anti-oxidant systems, free radicals and radical-free systems are pivotal to maintain the oxidation/reduction homeostasis and prevent oxidative stress.

Radical-free systems, that include thiol-oxidoreductases, are essential and abundant enzymatic systems that account for 0.5–1% of the cell proteome (Fomenko and Gladyshev, 2012). Many of these thiol-oxidoreductases form protein complexes where each thiol oxidoreductase frequently contains cysteine (Cys) as their conserved catalytic residue (Fomenko and Gladyshev, 2012). Thioredoxin-thioredoxin reductase (Trx/TrxR) and glutathione-glutathione reductase (GSH/GR) are the two main detoxifying systems independent of ROS.

The Trx/TrxR system has drawn significant attention due to its broad substrate specificity, allowing it to play important roles in regulating DNA synthesis, gene transcription, cell growth and apoptosis (Nozaki et al., 1999; Becker et al., 2000). Thioredoxin reductases (TrxRs) are enzymes that belong to the flavoprotein family of pyridine nucleotide-disulphide oxidoreductases (Mustacich and Powis, 2000). These enzymes are homodimeric proteins where each monomer contains a FAD domain, a NADPH binding domain, and an active site containing a redox active disulfide (Mustacich and Powis, 2000). Cysteine is present in the catalytic redox active center, which is highly conserved in thiol-reductases. The majority of thiol- oxidoreductases have a single catalytic Cys, but some of these enzymes are composed of two or more thiol-oxidoreductase domains, each having the catalytic redox Cys (Fomenko and Gladyshev, 2012).

Two main types of TrxRs (McMillan et al., 2009) are recognized:

- High molecular weight (H-TrxR) enzymes that contain a redox active center (motif CXXXXC) in the FAD binding domain. H-TrxR is closely related to glutathione reductase (GR), trypanothione reductase (TryR), mercuric reductase (MerR) and lipoamide dehydrogenase (LipD) (Nozaki et al., 1999). There are two varieties of H-TrxR: (a) one that contains a selenocysteine at the penultimate position in the C-terminal interface domain: the mammalian form; (b) one where selenocysteine have been replaced by cysteine in the interface domain: the apicomplexan parasite form.
- Low molecular weight (L-TrxR) enzymes that contain a redox active disulfide (motif CXXC) in the NADPH domain. L-TrxRs are related to alkyl hydroperoxide reductase F52A (AhpF). It is present in bacteria, fungi, plants and some protozoan parasites including *Trichomonas vaginalis* (Nozaki et al., 1999; Williams et al., 2000; Coombs et al., 2004) and *Entamoeba histolytica* (Arias et al., 2007).

Despite only 20% primary sequence identity between H-TrxR and L-TrxR (Nozaki et al., 1999), they appear to have evolved from a common ancestor but developed independently. L-TrxR and H-TrxR are mutually exclusive, suggesting that they do not act synergistically (Nozaki et al., 1999).

Electrons are transferred from NADPH via FAD to the active-site disulphide of TrxR, which then reduces the substrate (thioredoxin). H-TrxR has 3 redox active centers, whereas the L-TrxR have 2 redox centers and the transfer of reducing equivalents requires a conformational change, in contrast to H-TrxR (Williams et al., 2000).

Trx/TrxR systems vary according to parasites subgroups. Among aerotolerant protozoans, the absence of GSH/GR makes Trx/TrxR a main member of their antioxidant systems. In fact, *E. histolytica, T. vaginalis* and *G. lamblia* possess a full thioredoxin system, consisting of thioredoxin (Trx), Trx peroxidase and a L-TrxR (Hughes et al., 2003; Coombs et al., 2004; Arias et al., 2007; Leitsch et al., 2009). In the Apicomplexan group, *Plasmodium* possesses both GSH/GR and Trx/TrxR redox systems. Its complete thioredoxin system comprises thioredoxin reductase (TrxR), different thioredoxins, thioredoxin-like proteins, and thioredoxin-dependent peroxidases (TPx) (Kawazu et al., 2001; Nickel et al., 2006; Kehr et al., 2010).

Genome sequencing of *T. brucei* (Berriman et al., 2005), *T. cruzi* (El-Sayed et al., 2005), and *L. major* (Ivens et al., 2005) revealed that trypanosomatids lack genes for GSH/GR and Trx/TrxR. While in most eukaryotic organisms these latter systems maintain the intracellular thiol redox homeostasis, trypanosomatids depend exclusively on trypanothione [N1,N8-bis(glutathionyl)spermidine; T(SH)2] (Fairlamb et al., 1985; Fairlamb and Cerami, 1992) and trypanothione reductase (TryR) to keep dithiols in a reduced form (Fairlamb and Cerami, 1992; Krauth-Siegel and Comini, 2008). TryR is the only enzyme that connects the NADPH- and the thiol-based redox systems in these parasites, and it is related to H-TrxR. As such, it shares many physical and chemical properties with GR. TryR has been biochemically characterized in *T. cruzi* (Krauth-Siegel et al., 1987), *Leishmania* (Cunningham and Fairlamb, 1995), and *T. brucei* (Sullivan et al., 1989; Jones et al., 2010).

### *Entamoeba histolytica* thioredoxin reductase

*E. histolytica* trophozoites are considered aerotolerant organisms. They can survive in an anaerobic environment such as that of the human gut. During tissue invasion, they are exposed to high levels of ROS. Earlier studies showed that it can tolerate up to 5% oxygen in the gas phase (Bruchhaus et al., 1997; Choi et al., 2005; Loftus et al., 2005). Hence, the parasite must have means to minimize damage caused by ROS produced by the host immune system. In contrast to most organisms, *E. histolytica* lacks both glutathione reductase activity and glutathione synthetic enzymes; therefore, it relies on TrxR to prevent, regulate and repair the damage caused by oxidative stress.

The *E. histolytica* genome has a single TrxR-encoding gene (EHI_155440) (EhTrxR). EhTrxR belongs to the low molecular weight TrxR family (L-TrxR). It is 964 bp in length, lacks introns and encodes a 314-amino-acid protein with a molecular mass of 33.7 kDa and a pI of 6.34. EhTrxR size and domain topology resembles *E. coli* TrxR. Both proteins have an active site dithiol/disulfide center (Cys-Ala-Thr-Cys for EcTrxR, Cys-Ala-Ile-Cys for EhTrxR). In EhTrxR, the Cys residues in the catalytic center correspond to Cys 140 and Cys 143. Sequence homology to other TrxRs is 21% identity to *E. coli* TrxR (L-TrxR), *Trichomonas vaginalis* (23%), *Trypanosoma cruzi* (27%), and *Homo sapiens* (36%) (Arias et al., 2007).

The catalytic mechanism of L-TrxR has been extensively characterized in *E. coli*. Spatially, the NADPH and FAD domains of *E. coli* ThrxR do not make close contact with the isoalloxazine ring of FAD. Its NADPH domain rotates 66° while the FAD domain remains fixed. Then, the bound NADPH moves into close contact with the FAD isoallaxazine ring that allows electron transfer to FAD and the active-site disulphide (Waksman et al., 1994).

EhTrxR demonstrates an unusually high level of NADPH oxidase activity, which is protective against molecular oxygen required for the survival of these aerotolerant, anaerobic organisms (Bruchhaus et al., 1998). EhTrxR exhibited NADPH oxidase activity with hyperbolic saturation kinetics for NADPH, and its estimated Km and Vmax values are 3.6 μM and 0.37 U/mg respectively (Arias et al., 2007). As described by Arias et al. (2007) EhTrxR is considered a true reductase of thioredoxin in that it is not able to transfer reducing equivalents directly to Ehp29 (peroxiredoxin). On the other hand, high intracellular levels of cysteine compensate for the lack of glutathione preventing auto-oxidation in highly reducing environments (Nozaki et al., 1999). Interestingly, despite the fact that neither thioredoxin nor thioredoxin reductase configurations include a transmembrane hydrophobic domain, *E. histolytica* thioredoxin-thioredoxin reductase system was primarily located in the plasma membrane (Arias et al., 2008) without evidence of intracytoplasmic presence.

Besides ROS, *E. histolytica* is exposed to high concentrations of reactive nitrogen species (RNS) such as nitric oxide (NO) or S-nitrosothiols (such as GSNO and CySNO) during tissue invasion. Although high levels of these RNS might inhibit *E. histolytica* growth *in vitro* (Arias et al., 2012), the parasite is able to survive and multiply during tissue invasion. This suggests that *E. histolytica* detoxification system is versatile enough to tolerate hostile environments. This versatility was recently demonstrated by *E. histolytica* Trx-TrxR system ability to reduce RNS and use an alternative electron donor such as NADH (Arias et al., 2012).

As *E. histolytica* lacks glutathione, Cys is its major intracellular low molecular mass thiol (Nozaki et al., 1999) that can also react with NO to generate CysSNO. This metabolite is considered critical for S-nitrosylation (addition of NO to the thiol group in Cys) or S-thiolation (addition of Cys to another Cys thiol group) of cellular proteins (Arias et al., 2012). These metabolites can be reduced by EhTrxR as was demonstrated by *in vitro* assays where NADPH and EhTrxR were exposed to different concentrations of CySNO and GSNO. The rates of NADPH oxidation were increased proportionally, suggesting that these compounds (CySNO or GSNO) can be reduced in a reaction catalyzed by EhTrxR. The EhTrxR/Trx system reduces S-nitrosothiols (Arias et al., 2012) compounds and metronidazole (Leitsch et al., 2007) as well as interacts with downstream peroxidases that are critical for cellular redox homeostasis (Schlosser et al., 2013).

Unlike other thioredoxin reductases, EhTrxR does not exhibit high specificity for NADPH. Arias et al. (2012) evaluated the reduction of DTNB by EhTrxR using NADPH or NADH as electron donors. Although EhTrxR affinity for NADH is 10 times lower than that for NADPH, the enzyme activity with NADH is not negligible when compared to other thioredoxin reductases that exhibit high specificity toward NADPH (Arias et al., 2012). These results suggest that EhTrxR can use either NADPH or NADH as its reduced co-factor. This evidence strongly shows the versatility of EhTrx and its ability to protect the parasite from the ROS and RNI byproducts during host invasion. Altogether, EhTrxR properties make it an ideal drug target. In fact, metronidazole decreases EhTrxR reductase activity, by forming covalent adducts with this enzyme.

### *Giardia lamblia* thioredoxin reductase

*Giardia lamblia* (synonyms: *G. intestinali*s or *G. duodenalis*) is a flagellate whose trophozoites live in the small intestine, while the infectious cysts are shed in feces and survive outside the host. Giardia cysts can infect or re-infect humans, and can be transmitted in food, water or fomites since they are resistant to environmental conditions and chemicals. Giardia is considered an aerotolerant organism as even its cyst is able to take up oxygen, although only at 10–20% of the level of trophozoites (Paget et al., 1993).

*G. lamblia* has a genome size of approximately 10–12 Mb divided among five chromosomes (Adam, 2001). Giardia isolates are divided into eight assemblages (genotypes) from A to H. Assemblages A and B are the only ones typically associated with human infections (Adam, 2001). Both human Giardia assemblages A and B possess a single TrxR-encoding gene (GL 50803_9827 and GL50581_832). This single gene is 945 bp in length, lacks introns and encodes a 314 amino acid protein (GLTrxR) with a molecular mass of approximately 33.8 kDa and a pI of 6.59.

Like most aerotolerant protozoans*, G. lamblia* lacks mitochondria, superoxide dismutase, catalase, glutathione-glutathione reductase system but possesses a thioredoxin-thioredoxin reductase system while cysteine is its major low molecular weight thiol (Brown et al., 1998). Given the lack of ROS-dependent anti-oxidant systems and glutathione, the thioredoxin-thioredoxin reductase system is thought to constitute a major part of the antioxidant defense in this organism. It consists of a soluble dimeric FAD containing NADPH-dependent disulfide reductase, which contains a two 35-kDa subunits and a partially purified 12-kDa protein; a putative thioredoxin, a low molecular weight thioredoxin reductase (TrxR), which has 75% identity to the *E. coli* thioredoxin reductase (Brown et al., 1998), and a thioredoxin- peroxidase. All genes are expressed in trophozoites and cysts, although transcripts of most genes are present at lower levels in cysts (Faghiri and Widmer, 2011). Since Giardia cysts can take up oxygen (Paget et al., 1993), the Giardia thioredoxin-thioredoxin reductase system must also be important to maintain oxidation/reduction homeostasis in its trophozoites and cyst forms, making it an attractive drug target. This target is even more important given that metronidazole, a drug used in the treatment of giardiasis, had no detectable effect on oxygen uptake or viability in cysts due to impermeability of the *Giardia* cysts to metronidazole (Paget et al., 1993).

### Thioredoxin reductase-gold interactions: an unexploited drug target

Despite the huge public health burden of parasites worldwide, the paucity of available anti-parasitic drugs is striking. Reprofiling of old FDA approved drugs has become an alternative expeditious approach to drug discovery. Its advantage relies on already known drug safety and pharmacokinetic effects in humans that make them readily available for off-label indications.

Auranofin, originally approved to treat rheumatoid arthritis, is now the leading compound for anti-parasitic treatment development. Indeed, we have recently demonstrated that auranofin has activity against trophozoites of *Entamoeba histolytica* (Debnath et al., 2012) and *Giardia lamblia* (Tejman-Yarden et al., 2013). Due to its activity shown in high throughput screening studies, *in vitro* and *in vivo* rodent models of colitis and liver abscesses, auranofin was granted Orphan Drug Status. Auranofin has also shown promising activity against metronidazole-resistant *Giardia lamblia* (Tejman-Yarden et al., 2013), *Plasmodium falciparum* (Sannella et al., 2008), *Schistosoma mansoni* (Angelucci et al., 2009), *Leishmania infantum* (Ilari et al., 2012), *Brugia malayi* and *Onchocerca* (Bulman et al., 2015). Thus, auranofin has the potential to become an alternative broad spectrum anti-parasitic agent.

#### Auranofin as an anti-parasitic agent

Auranofin was the first oral gold salt approved by the FDA to treat rheumatoid arthritis over 25 years ago. Its pharmacokinetic characteristics are distinct from those of gold sodium thiomalate, a chrysotherapy agent of systemic administration. (Blodgett, 1983; Blodgett et al., 1984). Despite its clinical use, auranofin's mechanism of action is poorly understood.

Kuntz et al. (2007) were the first ones to describe the effect of auranofin in *S. mansoni*. In *in vitro* assays, they demonstrated that auranofin rapidly killed juvenile and adult forms of *S. mansoni* (Kuntz et al., 2007). Their preliminary *in vivo* data correlated with their *in vitro* findings: mice infected with *S. mansoni* and treated with auranofin showed a 59 and 63% decrease in worm burden compared to control mice.

Additionally, auranofin strongly inhibits the growth of malarial parasite *Plasmodium falciparum* (Sannella et al., 2008), the pro-mastigote stage of *Leishmania infantum* (Ilari et al., 2012), the bloodstream and procyclic stages of *Trypanosoma brucei* (Lobanov et al., 2006) and the larval worms of *Echinococcus granulosus* (Bonilla et al., 2008). In all these studies, auranofin's anti-parasitic dose was in in the nanomolar range, which is achievable in patients with rheumatoid arthritis (approximately 5 μM serum blood levels) (Debnath et al., 2012).

Angelucci et al. further analyzed *S. mansoni* thioredoxin-glutathione reductase (TGR) crystal structure in the presence of auranofin (Angelucci et al., 2009). The structure revealed gold (I) rather than auranofin as an adduct between pairs of cysteines (Cys-Au-Cys) in two different sites and also bound to the proposed NADPH binding site of the reductase in a third location.

Similarly, the crystal structure of reduced *Leishmania infantum* trypanothione reductase complexed with NADPH and auranofin also demonstrated that gold binds two active cysteine residues of trypanothione reductase (TryR) (Ilari et al., 2012), i.e., Cys52 and Cys57, while the thiol sugar moiety of auranofin binds to the trypanothione binding site; thus auranofin appears to inhibit TryR through a dual mechanism.

**Figure 1:**
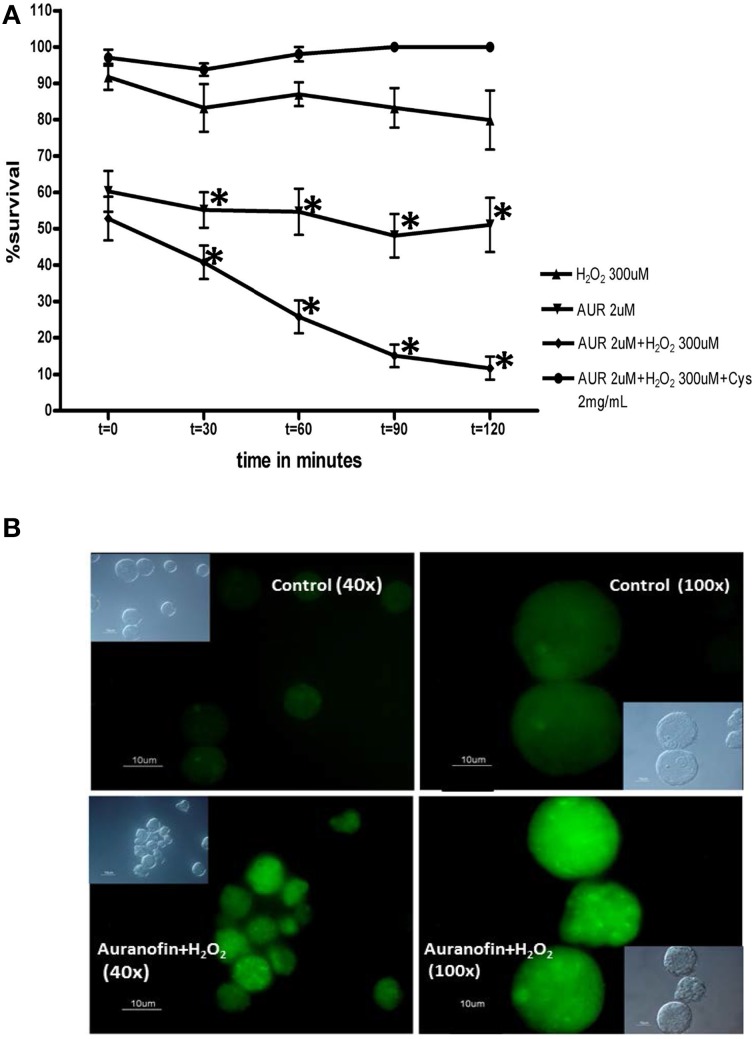
**(A)** *In vitro* susceptibility of trophozoites (control and treated with auranofin) to reactive oxygen species (H_2_O_2_) and the effect of added cysteine. Time points with mean ± SEM % survival. Experiments are shown in triplicates. ^*^p < 0.002 by Student's *t*-test. **(B)** Immunoflouroscence microscopy: Detection of reactive oxygen species within *E. histolytica* trophozoites following treatment with auranofin or auranofin plus H_2_O_2_. Control trophozoites were treated with ethanol alone (auranofin carrier). Insets are differential interference contrast images. Scale bars, 10 μm. Figures were first published in Andrade and Reed (2015).

**Figure 2:**
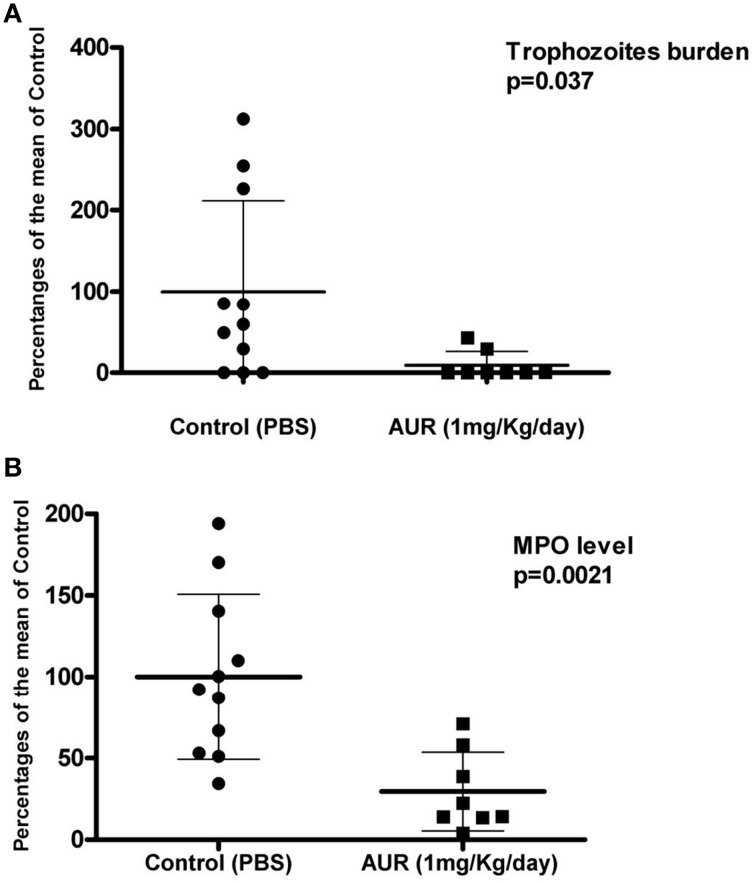
**Experimental mice model of amebic colitis**. Control and treatment with auranofin groups (*n* = 8) are presented as: **(A)** The percentage of trophozoites per gram of tissue. **(B)** Or myeloperoxidase (MPO) units per gram of tissue compared with the means of infected controls (as 100%).

We recently demonstrated that auranofin has activity against *E. histolytica* trophozoites (Debnath et al., 2012). Our studies support the hypothesis that EhTrxR likely is the target for auranofin. Our qualitative *in vitro assays* showed that *E. histolytica* trophozoites treated with auranofin were more susceptible to oxidative stress in a time dependent manner (Figure 1A). Staining *E. histolytica* trophozoites with dichloro-dihydrofluorescein, which fluoresces green upon contact with ROS, showed that trophozoites treated with auranofin accumulated more ROS (Debnath et al., 2012) (Figure 1B). This effect was inhibited by cysteine, the major reductant in *E. histolytica*, which protected trophozoites that were treated with auranofin (Figure 1). Our *in vitro* findings were corroborated in animal models of colitis and liver abscesses where auranofin not only decreased the parasite tissue burden, but also the local inflammatory response as measured by activity of myeloperoxidase in cecal tissue (Debnath et al., 2012) (Figures 2A,B respectively). Besides its activity against trophozoites, auranofin seems to have activity against cysts of *E. invadens*, the only ameba to readily encyst *in vitro* (R. Andrade, manuscript in preparation).

*G. lamblia* assemblages A and B were also susceptible to auranofin, as demonstrated by Tejman-Yarden et al. (2013). Auranofin displayed a half-maximal effective concentrations (EC50s) of 4–6 μM at 48-h. Most importantly, auranofin was able to overcome resistance to metronidazole. In fact, the EC50 for auranofin was not significantly different between metronidazole- sensitive parental *Giardia* isolates and several of their metronidazole-resistant isogenic derivative lines.

Similarly, to *E. histolytica* and *G. lamblia*, auranofin was found effective against *C. parvum in vitro* with EC50 about 2 μM, which was comparable to nitazoxanide, the current drug of choice (Debnath et al., 2013).

Altogether, these findings suggest that auranofin may be a new promising broad spectrum antiparasitic agent with activity against ameba, giardia and cryptosporidium. Currently, clinical trials using auranofin to treat ameba and giardia infections are about to begin.

## Funding

This publication was supported in part by a Harold Amos Minority Medical Faculty Development Program (RMA), UCSD Academic Senate Grant 70642 (RMA), NIH 2T32AI007036-31A1 (RMA), NIH grant 5U01AI077822-04, and U01 AI110435 (SLR). The funders had no role in study design, data collection and analysis, decision to publish, or preparation of the manuscript.

### Conflict of interest statement

The authors declare that the research was conducted in the absence of any commercial or financial relationships that could be construed as a potential conflict of interest.

## Footnotes

^1^http://www.cdc.gov/parasites/giardia/epi.html. July 13, 2012

^2^http://www.cdc.gov/mmwr/preview/mmwrhtml/ss6105a1.htm

^3^http://www.niaid.nih.gov/topics/BiodefenseRelated/Biodefense/Pages/CatA.aspx
